# *Burkholderia lata* Infections from Intrinsically Contaminated Chlorhexidine Mouthwash, Australia, 2016

**DOI:** 10.3201/eid2411.171929

**Published:** 2018-11

**Authors:** Lex E.X. Leong, Diana Lagana, Glen P. Carter, Qinning Wang, Kija Smith, Tim P. Stinear, David Shaw, Vitali Sintchenko, Steven L. Wesselingh, Ivan Bastian, Geraint B. Rogers

**Affiliations:** South Australian Health and Medical Research Institute, Adelaide, South Australia, Australia (L.E.X. Leong, S.L. Wesselingh, G.B. Rogers);; Flinders University, Bedford Park, South Australia, Australia (L.E.X. Leong, G.B. Rogers);; Royal Adelaide Hospital, Adelaide (D. Lagana, D. Shaw);; University of Melbourne, Melbourne, Victoria, Australia (G.P. Carter, T.P. Stinear);; The University of Sydney, Westmead, New South Wales, Australia (Q. Wang, V. Sintchenko);; SA Pathology, Adelaide (K. Smith, I. Bastian)

**Keywords:** *Burkholderia lata*, chlorhexidine mouthwash, intensive care unit, genome analysis, bacteria, nosocomial infections, Australia

## Abstract

*Burkholderia lata* was isolated from 8 intensive care patients at 2 tertiary hospitals in Australia. Whole-genome sequencing demonstrated that clinical and environmental isolates originated from a batch of contaminated commercial chlorhexidine mouthwash. Genomic analysis identified efflux pump–encoding genes as potential facilitators of bacterial persistence within this biocide.

*Burkholderia contaminans* and *B. lata* together form group K of the *B. cepacia* complex (Bcc). These predominantly environmental species are a major cause of pharmaceutical contamination and have been linked to multiple instances of associated opportunistic infection (*1*). Although both species are capable of causing serious infections in humans (*2*,*3*), only *B. contaminans* has been associated with infection outbreaks (*3*,*4*). We report a healthcare-associated *B. lata* infection outbreak occurring in intensive care units (ICUs) in 2 tertiary hospitals in Australia.

During May–June 2016, bacterial contamination of chlorhexidine mouthwash (0.2% mg/mL) was associated with an interjurisdictional outbreak in New South Wales and South Australia. Bcc isolates were obtained from blood and tracheal aspirates from 6 ICU patients in hospital A (South Australia) (sample information and isolation protocols detailed in the Technical Appendix). An investigation by the hospital’s infection and prevention control team noted discoloration of a commercial chlorhexidine mouthwash. Bcc isolates were cultured from all 5 tested bottles from the discolored batch but not from 11 bottles tested from 4 other batches. Isolates were further obtained from the surfaces of hand basins in 3 separate ICU rooms, where bottles of mouthwash from the contaminated batch were in use. Separately, a Bcc isolate was obtained from the sputum of an ICU patient in hospital B (New South Wales), where the same batch of mouthwash was in use. After a nationwide recall of the contaminated mouthwash batch, no further cases were reported. 

The genomes of 15 Bcc isolates from the 2 hospitals were sequenced (Technical Appendix). A closed genome of isolate A05 was generated as a reference, consisting of 3 circular chromosomes of 3.93, 3.71, and 1.15 Mbp (National Center for Biotechnology Information BioProject accession no. PRJNA419071).

Genome analysis identified an Australasian sublineage of *B. lata* as the cause of the outbreak. Specifically, all isolates were sequence type 103 (ST103), which sits within a subclade of *B. lata* isolates from Australia and New Zealand (Technical Appendix Figure 1). Two isolates from hand basins (A07 and A08) and 1 from a hospital bench (A10) were of unknown sequence type.

Single-nucleotide polymorphism (SNP)–based phylogenetic analysis identified the outbreak strain as a distinct clonal lineage (0–1 SNPs) within the group K clade (Figure), separated from other members of the group by a minimum of 167,662 SNPs. SNP variation within the clonal sublineage ranged from 0 to 2 SNPs across isolates from contaminated mouthwash bottles and patients. A single SNP distinguished isolates from patient 4 in room 21 (A04) and the hand basin in the same room (A08) (collected 2 days apart). Three of the taxa isolated from the hospital environment (A07, A08, and A10) had substantial SNP variations compared with contaminated mouthwash and patient isolates (an average of 191,893 SNPs for A07, 655 SNPs for A08, and 1,408 SNPs for A10).

**Figure:**
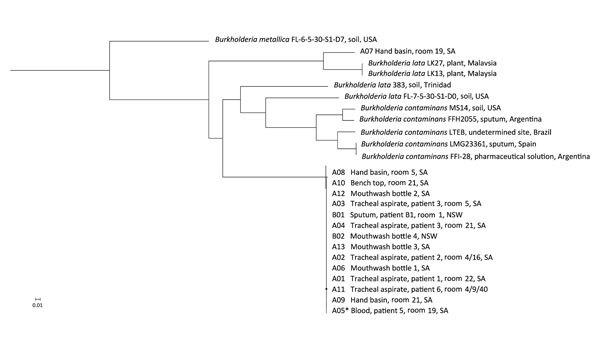
Phylogenetic analysis of isolates implicated in an outbreak *Burkholderia lata* infection from intrinsically contaminated chlorhexidine mouthwash, Australia, 2016. The maximum-likelihood tree is constructed from core genome single-nucleotide polymorphism alignments (N = 512,480) of the outbreak genomes, bootstrapped 1,000 times, and archival genomes from *B. cepacia* complex group K, relative to the reference genome *B. lata* A05 (identified by an asterisk). *B. metallica* was included as a comparator.

Differences of 0–1 SNPs between isolates from mouthwash in the 2 hospitals, including from unopened bottles, strongly suggests contamination during the manufacturing process. After several previous incidents of contamination of cosmetic, toiletry, and pharmaceutical products by members of Bcc, the US Food and Drug Administration commented that these bacteria very likely are introduced into the manufacturing processes through contaminated water (https://www.fda.gov/Drugs/DrugSafety/ucm559508.htm). Direct patient-to-patient transmission of *B. lata* would appear unlikely in this instance, given the use of individual ICU rooms with nonshared facilities and incomplete overlap in periods of care.

Chlorhexidine mouthwash is perhaps a surprising source of *B. lata* infection, given its potent biocidal properties. However, Bcc species have been isolated previously from 0.02% chlorhexidine gluconate (in irrigation apparatuses used for vaginal douching) and from 0.05% preparations (used for storage of suction catheters) (*5*). The ability of *B. lata* to remain viable in chlorhexidine appears to be a result of a combination of efflux pump activity, biofilm formation, and cell wall impermeability (*1*,*6*,*7*). These mechanisms probably also contribute to the common inefficacy of combination antibiotic therapy in the treatment of associated infections (*8*). Prolonged exposure to microbiocides, including chlorhexidine, has been shown to result in a stable increase in the expression of antibiotic-resistance mechanisms (*1*,*6*), and elevated chlorhexidine resistance has been reported in multidrug-resistant strains of *B. cenocepacia* from cystic fibrosis patients (*9*). Three resistance-nodulation-division (RND) efflux pump genes (RND3, RND4, and RND9) have been shown to be essential for chlorhexidine tolerance in *B. cenocepacia* (*9*). Examination of the complete genome of *B. lata* isolate A05 revealed the presence of RND3, RND4, and RND9 in each strain (>94% sequence identity) (Technical Appendix Figure 2).

*B. contaminans* is the cause of widespread pharmaceutical product contamination, and infection outbreaks by this species are well-documented (*3*,*10*). Our findings suggest that the other member of Bcc group K, *B. lata*, also represents an important opportunistic pathogen of relevance to infection control, particularly given its intrinsic biocide tolerance.

Technical Appendix Materials and methods for investigation of *Burkholderia lata* infections from intrinsically contaminated chlorhexidine mouthwash, Australia, 2016.
