# Supplementary material for: Burkholderia lata Infections from Intrinsically Contaminated Chlorhexidine Mouthwash, Australia, 2016
Source: Emerg Infect Dis. 2018 Nov;24(11):2109–11. doi: 10.3201/eid2411.171929 (PMC6199994; doi:10.3201/eid2411.171929)
Supplement: Technical Appendix — Materials and methods for investigation of Burkholderia lata infections from intrinsically contaminated chlorhexidine mouthwash, Australia, 2016. [file 17-1929-Techapp-s1.pdf]

Article DOI: https://doi.org/10.3201/eid2411.171929

Burkholderia lata Infections from Intrinsically Contaminated Chlorhexidine Mouthwash, Australia, 2016

Technical Appendix

Materials and Methods

Sample Collection

Patient information is shown in Technical Appendix Table. Isolates from Hospital A and Hospital B were recovered from clinical samples by SA Pathology (Adelaide, South Australia) and by NSW Health Pathology (Sydney, New South Wales), respectively. Bcc isolates from the contaminated chlorhexidine mouthwash and ICU patients were cultured using Blood agar (under CO_2 and anaerobic conditions), Chocolate agar, and Nalidixic acid colistin agar. Environmental samples (hand-basins and bench tops) were collected using liquid amies flocked swabs and cultured on Blood, MacConkey and *Burkholderia cepacia* selective agar.

Genomic DNA Isolation and Sequencing

Genomic DNA from Hospital A and Hospital B isolates was extracted and sequenced on an Illumina NextSeq500 platform (Illumina Inc, California, USA). A closed genome was obtained from isolate A05 through Single Molecule, Real-Time (SMRT) sequencing on a PacBio RS II platform using P6-C4 chemistry (Pacific Bioscience Inc, California, USA) according to the 20 kb template preparation using BluePippin size-selection protocol (Pacific Bioscience).

Genomic Analysis

Illumina sequencing reads were processed using the Nullarbor pipeline (https://github.com/tseemann/nullarbor). Single-nucleotide polymorphism (SNP) analysis was performed using Snippy v3.1 (https://github.com/tseemann/snippy) with isolate A05 as a reference. The reference genome was assembled as described with indels corrected using Illumina sequencing reads (*1*). A maximum-likelihood phylogenetic tree was created based on the SNP variations in the core genome using PhyML v3.1 (*2*), with complete and draft genomes

from other Bcc group K strains providing context (*B. lata* strain 383, *B. lata* LK27, *B. lata* LK13, *B. lata* FL-7–5-30-S1-D0, *B. contaminans* MS14, *B. contaminans* FFH2005, B. contaminans LTEB, *B. contaminans* LMG23361, *B. contaminans* FFI-28, and *B. metallica* FL-6–5-30-S1-D7). Genes encoding putative efflux pump within the *B. cenocepacia* J2315 genome were used to identify orthologous sequences within Bcc group K (E-value $\leq 10^{-5}$). Multi loci sequence typing (MLST) analysis was performed using MLST (https://github.com/tseemann/mlst, version 2.0). To construct a maximum likelihood tree for MLST results, allelic sequences for all Bcc isolates were retrieved from BIGSdb database (*3*). Orthologous sequences in B. lata isolate genomes were identified using HMMer v.3.1b2, aligned to database sequences using MUSCLE (v3.8.1551), and concatenated.

References

1. Baines SL, Howden BP, Heffernan H, Stinear TP, Carter GP, Seemann T, et al. Rapid emergence and evolution of *Staphylococcus aureus* clones harboring *fusC*-containing staphylococcal cassette chromosome elements. Antimicrob Agents Chemother. 2016;60:2359–65. PubMed http://dx.doi.org/10.1128/AAC.03020-15

2. Guindon S, Gascuel O. A simple, fast, and accurate algorithm to estimate large phylogenies by maximum likelihood. Syst Biol. 2003;52:696–704. PubMed http://dx.doi.org/10.1080/10635150390235520

3. Jolley KA, Maiden MCJ. BIGSdb: Scalable analysis of bacterial genome variation at the population level. BMC Bioinformatics. 2010;11:595. PubMed http://dx.doi.org/10.1186/1471-2105-11-595

Technical Appendix Table. Outbreak strains of *Burkholderia lata* isolated from clinical samples, contaminated mouthwash bottles and ICU sinks and bench top in two Australian tertiary hospitals

Hospital	Isolate	Room	Specimen	Patient	Initial presentation	Isolation Date
Clinical isolates						
A	01	22	Tracheal aspirate	A1	Pneumonia	15 May 2016
A	02	4,16	Tracheal aspirate	A2	Dissecting aortic aneurysm	14 June 2016
A	03	5	Tracheal aspirate	A3	Aortic dissection	18 June 2016
A	04	21	Tracheal aspirate	A4	Intracranial injury	22 June 2016
A	05	19	Blood	A5	Statin myositis	7 May 2016
A	11	4,9,40	Tracheal aspirate	A6	Angina	26 June 2016
B	01	1	Sputum	B1	Pneumonia	24 June 2016
Nonclinical isolates						
A	06		Mouthwash bottle 1			25 June 2016
A	07	19	Hand basin			20 May 2016
A	08	5	Hand basin			24 May 2016
A	09	21	Hand basin			24 June 2016
A	10	21	Bench top			27 June 2016
A	12		Mouthwash bottle 2			29 June 2016
A	13		Mouthwash bottle 3			29 June 2016
B	02		Mouthwash bottle 4			4 July 2016

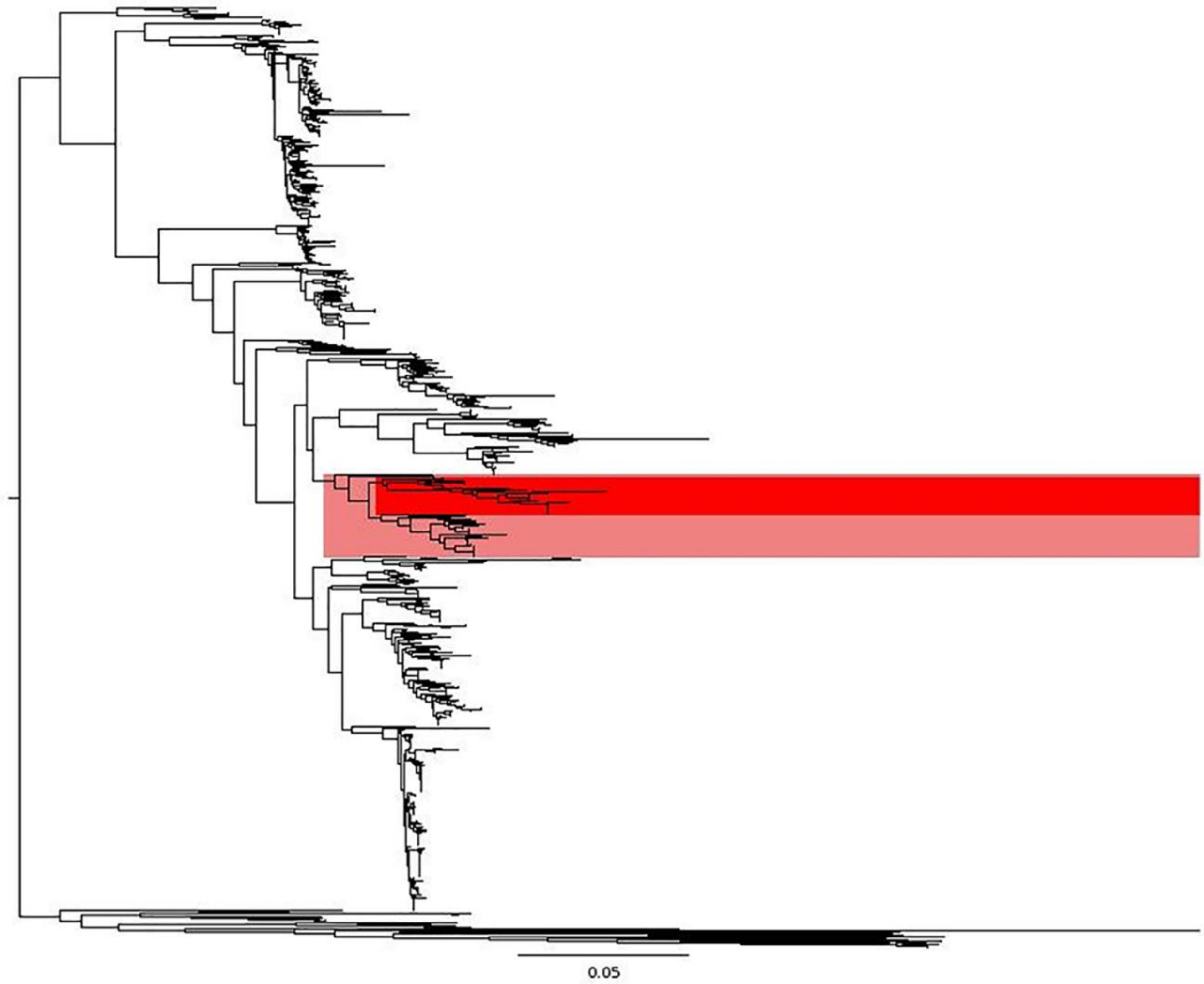

Technical Appendix Figure 1. MLST phylogenetic tree of the outbreak *Burkholderia lata* from Australasian sublineage. MLST allelic sequences for the all *Burkholderia cepacia* complex were obtained from PUBMLST database {Jolley, 2010 #324}. Aligned allelic sequences were concatenated and maximum likelihood tree was computed using PhyML v3.0 {Guindon, 2010 #325} with parameters of HKY85 as model of nucleotide substitution and BioNJ as initial tree. Group K of the BCC were highlighted in light red, and *B. lata* taxa were highlighted in dark red.

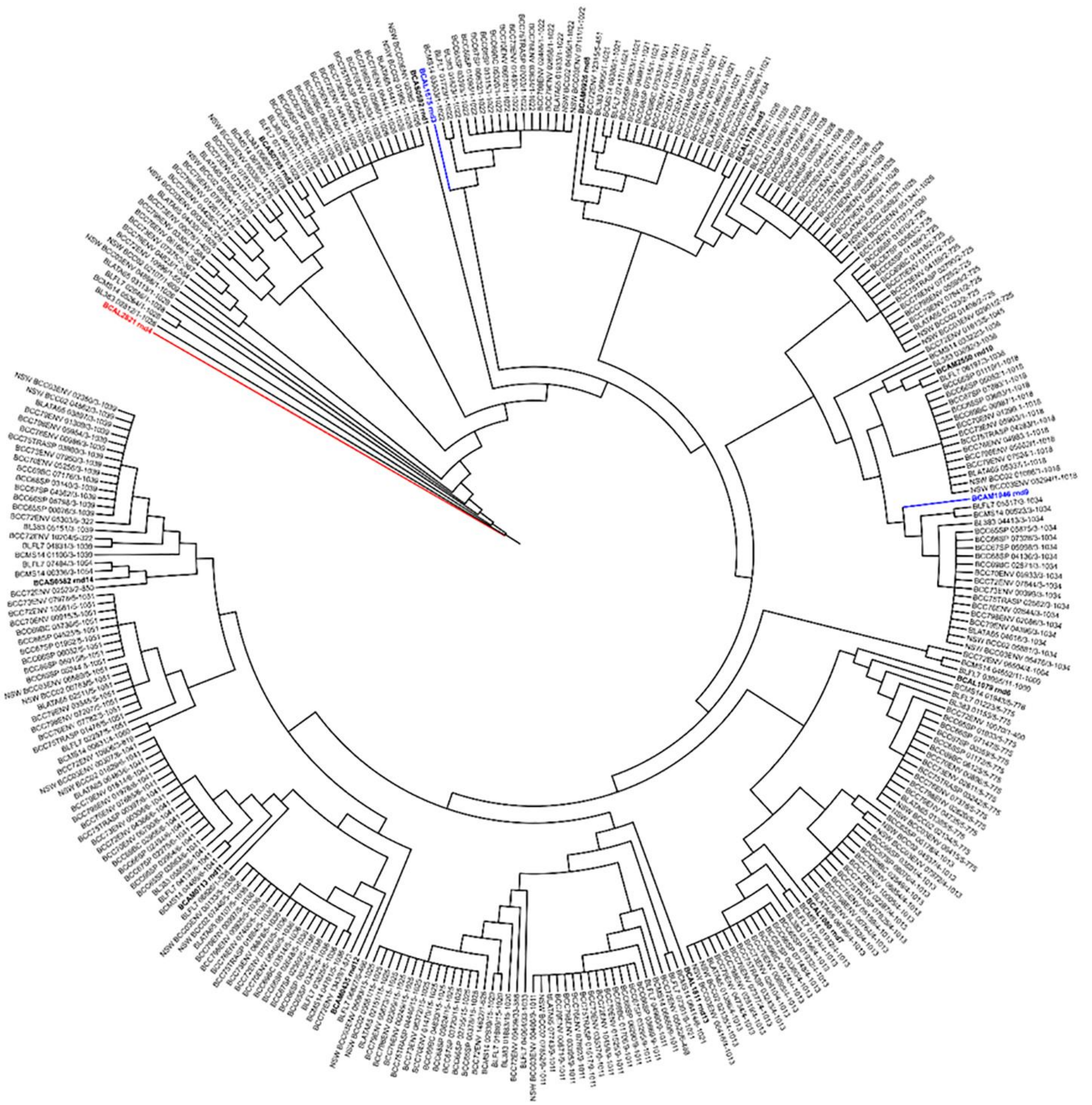

Technical Appendix Figure 2. Maximum likelihood tree for the bacterial multidrug efflux pump (RND) proteins of the Bcc Group K. The tree was built with LG as amino acid substitution model against the efflux pumps of *B. cenocepacia* J2135 (highlighted in bold, with RND 3 and 9 in blue, while RND 4 is in red).
